# Coixol Suppresses NF-κB, MAPK Pathways and NLRP3 Inflammasome Activation in Lipopolysaccharide-Induced RAW 264.7 Cells

**DOI:** 10.3390/molecules25040894

**Published:** 2020-02-18

**Authors:** Yusheng Hu, Qilyu Zhou, Tianlong Liu, Zhongjie Liu

**Affiliations:** 1 College of Biological Sciences, China Agricultural University, Beijing 100193, China; hys_test@163.com; 2 College of Veterinary Medicine, China Agricultural University, Beijing 100193, China; zhou7l@163.com (Q.Z.); liutianlong@cau.edu.cn (T.L.)

**Keywords:** coix, polyphenol, coixol, anti-inflammatory, NF-κB pathway, MAPK pathway, NLRP3 inflammasome

## Abstract

Coixol, a plant polyphenol extracted from coix (*Coix lachryma-jobi* L.var.*ma-yuen* Stapf), has not been investigated for its anti-inflammatory effect. In this study, using a lipopolysaccharide (LPS)-induced macrophage cell model, we observed that coixol can effectively reduce the expression of interleukin (IL)-1β, IL-6, IL-18, tumor necrosis factor (TNF)-α, nitric oxide (NO), inducible nitric oxide synthases (iNOS), and cyclooxygenase (COX)-2, but had no effect on the expression of the anti-inflammatory mediator IL-10. Furthermore, we found that coixol inhibits mitogen-activated protein kinases (MAPKs), nuclear transcription factor κ B (NF-κB) pathways, and NOD-like receptor protein (NLRP) 3 inflammasome activation. In conclusion, the present study demonstrates that coixol exerts certain anti-inflammatory effects by inhibiting the expression of pro-inflammatory mediators in vitro. The mechanism of this effect was in part related to its ability to inhibit the activation of NF-κB, MAPKs pathways, and NLRP3 inflammasome.

## 1. Introduction

Inflammation is a defensive response of the body to injury. The source of injury can be exogenous or endogenous, and almost all disease processes are accompanied by inflammation. Studies have shown that inflammation plays an important role in the development of various diseases, such as ulcerative colitis and Crohn’s disease [1], arthritis [2], hypertension [3], depression [4], obesity [5], and even multiple cancers [6]. Because inflammation is one of the most basic immune responses, its process is usually well regulated. Macrophages are one of the key cells which regulate the inflammatory response.

Macrophages are the main component of the monocyte phagocyte system, which is formed by monocyte migration from the circulation to local tissues. Macrophages are activated when exposed to lipopolysaccharides (LPS), cytokines (including interleukin (IL)-1β, IL-6, tumor necrosis factor (TNF)-α, and interferon γ, among others) and other chemical agents [7]. Activated macrophages regulate the inflammatory process by phagocytosis, antigen presentation, and secretion of various cytokines. Research has shown that macrophages play a key role in the occurrence, maintenance, and regression of inflammation [8]. Therefore, macrophages are widely used as model cells in the research of anti-inflammatory drugs.

LPS is the cell wall component of Gram-negative bacteria. As an endotoxin, it can stimulate macrophages to secrete a variety of proinflammatory mediators, such as nitric oxide (NO), IL-1β, IL-6, and TNF-α, thus triggering a series of immune reactions in the body. The expression of these cytokines is closely related to the activation of the nuclear transcription factor κ B (NF-κB) pathway and its upstream mitogen-activated protein kinases (MAPKs) pathway [9,10]. In addition, the NLRP3 inflammasome, which mediates pyroptosis, can also mediate the inflammatory response by promoting the secretion of IL-1β [11].

Coix (*Coix lachryma-jobi* L.var.*ma-yuen* Stapf) originated from South Asia and was widely planted in Asian countries as food and medicine [12]. In traditional Chinese medicine (TCM), coix seed has diuretic, analgesic, antiemetic and other effects [13], which are commonly used to treat gastrointestinal diseases, skin diseases [14], even tumors [15]. In recent years, polyphenols have been the research hotspot of coix compounds [16]. Coixol is a polyphenolic compound extracted from coix, but its regulatory effect on inflammation has not been investigated. Many coix extracts have been reported to have anti-inflammatory effects [17]. In the light of reports on the anti-inflammatory effect of plant polyphenols [18], we speculated that coixol has certain anti-inflammatory effects.

The aim of this study was to evaluate the anti-inflammatory effect of coixol on the expression of various inflammatory mediators in an in vitro model (LPS-induced RAW 264.7 cells). Furthermore, the phosphorylation of p65 and inhibitors κB (IκB) was used to observe the activation of the NF-κB pathway. The activation of MAPK pathways was assessed by examining the phosphorylation of extracellular-signal-regulated protein kinase (ERK), c-Jun amino-terminal kinase (JNK), and p38. In addition, the expression of NOD-like receptor protein (NLRP) 3, apoptosis-associated speck-like protein containing CARD domain (ASC), and caspase-1 were examined in order to observe the activation of NLRP3 inflammasome. Through the investigation of NF-κB, MAPK pathways and NLRP3 inflammasome, we hope to reveal the possible mechanism of its anti-inflammatory effect.

## 2. Results

### 2.1. Cytotoxic Effects of Coixol on RAW 264.7 Cells

**Figure 1:**
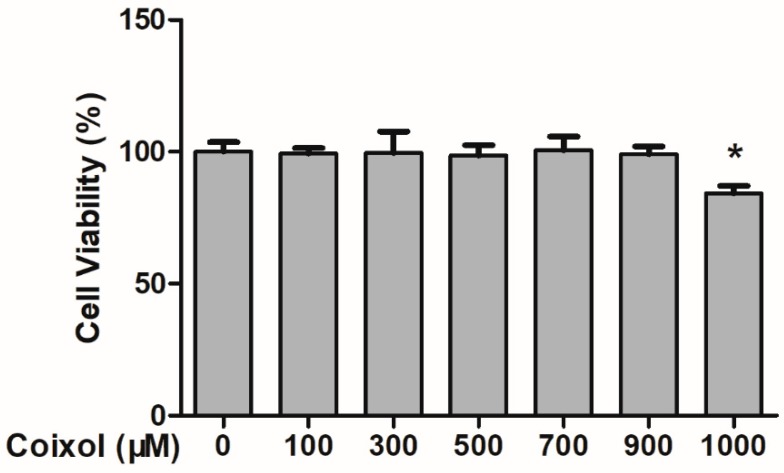
Effects of coixol on cell viability in RAW 264.7 cells. Cells were treated with various concentrations of coixol for 8 h. Cell viability was measured using a Cell Counting Kit-8. The data represent the mean ± SD (*n* = 6). * *p* < 0.05 vs. control group.

As shown in Figure 1, coixol had no cellular toxicity at concentrations of up to 900 μmol/L over a period of 8 h. Therefore, concentrations 400 and 800 μmol/L were selected for subsequent testing.

### 2.2. Production of IL-1β, IL-6, TNF-α, and IL-10

**Figure 2:**
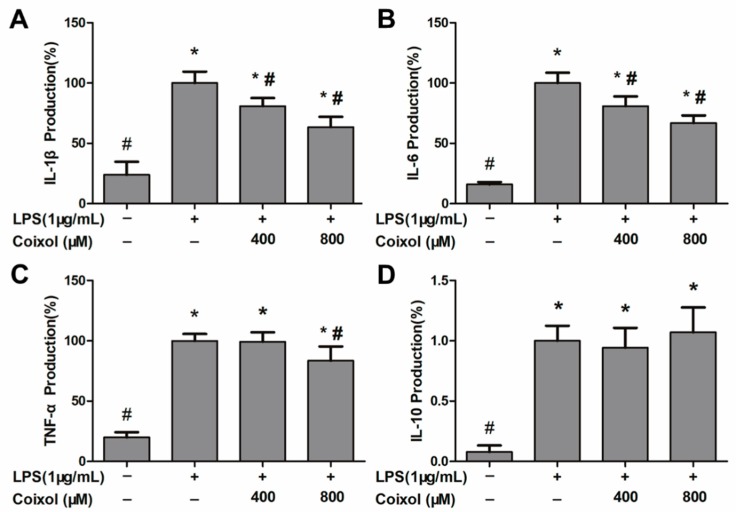
Effects of coixol on the production of cytokines in LPS-induced RAW 264.7 cells supernatant. Cells were pretreated with coixol for 4 h and then stimulated with 1μg/mL LPS for 4 h. The productions of (**A**) interleukin (IL)-1β, (**B**) IL-6 and (**C**) tumor necrosis factor (TNF)–α and (**D**) IL-10. Cytokine productions were measured using an ELISA Kit. The data represent the mean ± SD (*n* = 6). * *p* < 0.05 vs. control group. # *p* < 0.05 vs. LPS group.

ELISA was used to detect the expression of inflammatory cytokines (IL-1β, IL-6, TNF-α, and IL-10) in the supernatant of cells. As shown in Figure 2A,B, coixol significantly reduced the overexpression of IL-1β and IL-6 induced by LPS stimulation in a dose-dependent manner. However, only a high concentration of coixol inhibits the upregulation of TNF-α expression (Figure 2C). For IL-10, coixol was unable to change the overexpression induced by LPS (Figure 2D).

### 2.3. Expression of NO, iNOS, and COX-2

**Figure 3:**
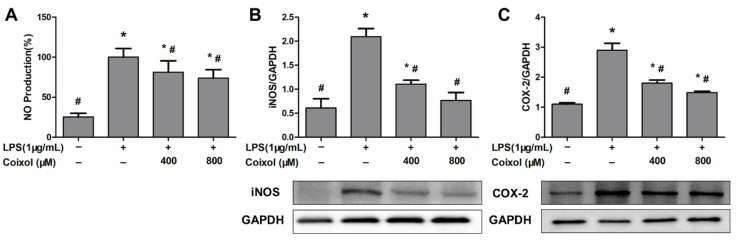
Effects of coixol on the expression of proinflammatory mediators in LPS-induced RAW 264.7 cells. Cells were pretreated with coixol for 4 h and then stimulated with 1μg/mL LPS for 4 h. (**A**) The production of nitric oxide (NO) in cells supernatant. NO was measured by Griess assay. The protein levels of (**B**) inducible-nitric oxide synthase (iNOS) and (**C**) cyclooxygenase (COX)-2. Protein levels were measured by western blotting. GAPDH was used as internal control. The data represent the mean ± SD (NO, *n* = 6; iNOS, COX-2, *n* = 3). * *p* < 0.05 vs. control group. # *p* < 0.05 vs. LPS group.

Coixol significantly reduced the LPS-induced upregulation of NO production, but there was no significant difference in NO production between different concentrations of coixol (Figure 3A). Similar to the production of NO, coixol inhibited the over-expression of iNOS and COX-2 caused by LPS, but there was no significant difference in the effect of different concentrations of coixol (Figure 3B,C).

### 2.4. Expression of NF-κB Pathways

**Figure 4:**
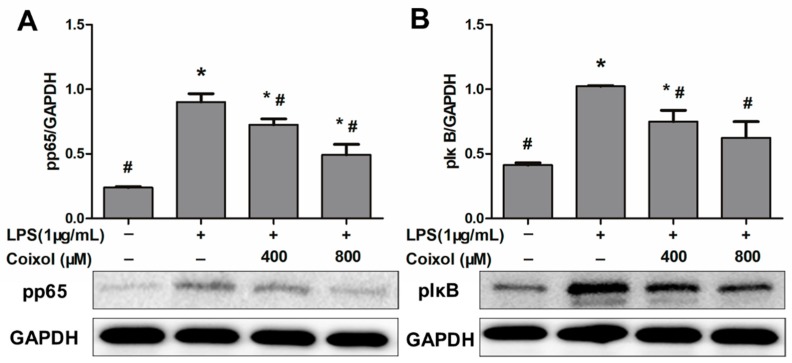
Effects of coixol on NF-κB signaling activation in LPS- induced RAW 264.7 cells. Cells were pretreated with coixol for 4 h and then stimulated with 1μg/mL LPS for 4 h. The protein levels of (**A**) phospho (p) p65 and (**B**) pIκB. Protein levels were measured by western blotting. GAPDH was used as internal control. The data represent the mean ± SD (*n* = 3). * *p* < 0.05 vs. control group. # *p* < 0.05 vs. LPS group.

In order to explore the possible mechanism of coixol-induced changes in RAW264.7 cells, the expression levels of key proteins (pIκB and pp65) in the NF-κB signaling pathway, which are closely related to inflammation, were detected by western blotting. As shown in Figure 4, coixol inhibited the over-expression of pp65 induced by LPS in a dose-dependent manner (Figure 4A); similarly, coixol also inhibited the over-expression of pIκB, but there was no significant difference in the inhibition effect of high concentration and low concentration coixol on the expression of pIκB (Figure 4B).

### 2.5. Expression of MAPK Pathways

**Figure 5:**
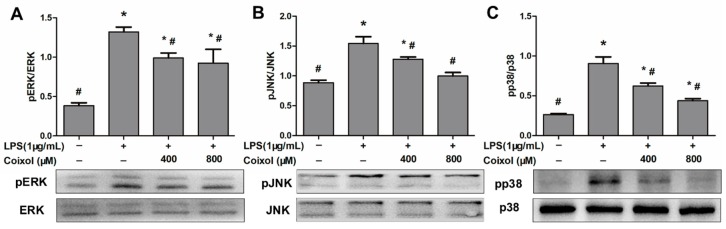
Effects of coixol on the activation of MAPK signaling in LPS-induced RAW 264.7 cells. Cells were pretreated with coixol for 4 h and then stimulated with 1 μg/mL LPS for 4 h. The protein levels of (**A**) phospho (p) ERK, (**B**) pJNK and (**C**) pp38. Protein levels were measured by western blotting. ERK, JNK, and p38 were used as internal control. The data represent the mean ± SD (*n* = 3). * *p* < 0.05 vs. control group. # *p* < 0.05 vs. LPS group.

MAPK pathways are recognized as closely related to the inflammatory response. Three classical MAPK signaling pathways (ERK, JNK, and p38) were detected in this study. The phosphorylation level of ERK was significantly reduced by the coixol, but there was no significant difference in the effects of different concentrations of coixol on ERK (Figure 5A). As shown in Figure 5B,C, Coixol downregulated the phosphorylation of JNK and p38 induced by LPS in a dose-dependent manner.

### 2.6. Expression of NLRP3 Inflammasome

**Figure 6:**
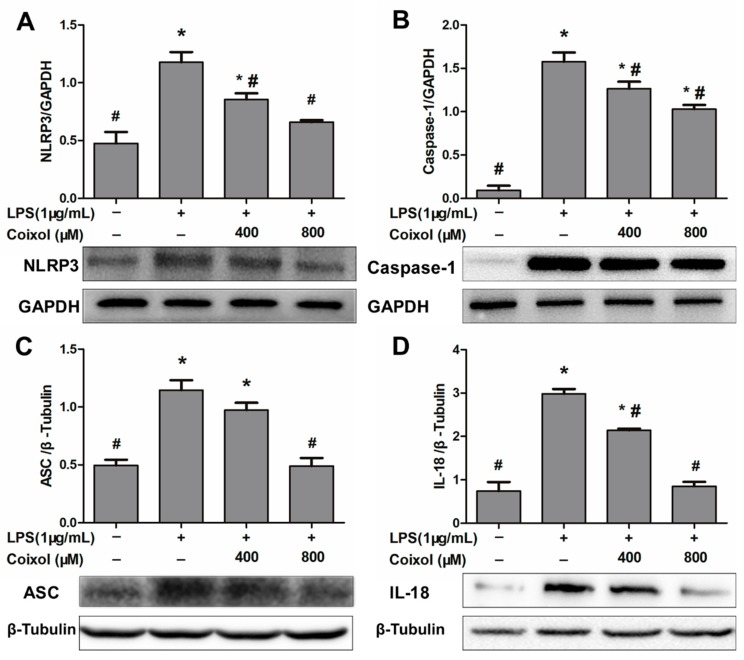
Effects of coixol on NLRP3 inflammasome activation in LPS- induced RAW 264.7 cells. Cells were pretreated with coixol for 4 h and then stimulated with 1μg/mL LPS for 4 h. The protein levels of (**A**) NOD-like receptor protein (NLRP) 3, (**B**) caspase-1, and (**C**) apoptosis-associated speck-like protein containing CARD domain (ASC) and (**D**) interleukin (IL)-18. Protein levels were measured by western blotting. GAPDH and β-Tubulin were used as internal controls. The data represent the mean ± SD (*n* = 3). * *p* < 0.05 vs. control group. # *p* < 0.05 vs. LPS group.

NLRP3 inflammasome is a protein complex related to inflammatory reactions, and its activation can promote the secretion of IL-1β and IL-18. In order to explore the mechanism of coixol-induced inhibition of IL-1β secretion, the component proteins of NLRP3 inflammasome (NLPR3, caspase-1, and ASC) and the IL-18 were examined by western blotting. As shown in Figure 6, coixol reduced the expression of NLRP3, but there was no significant difference between high dose and low dose (Figure 6A). Caspase-1 expression was also down-regulated in a dose-dependent manner (Figure 6B). Only the high concentration of coixol was able to down-regulate ASC expression (Figure 6C), whereas coixol down-regulated the expression of IL-18 induced by LPS in a dose-dependent manner.

## 3. Discussion

Coixol is an extract from coix and has been studied for its bioactivity since the 1980s [19]. The reported biological activities of coixol include but are not limited to the regulation of reproductive behavior and hormones [20], antimicrobial and antitumor activity [21,22], inhibition of mucin secretion [23], and enhancement of insulin secretion [24]. The anti-inflammatory effect of coixol has not been reported, although some other extracts of coix have shown anti-inflammatory activity [17,25].

In this study, we evaluated the response of macrophages to the stimulation by LPS, because macrophages play an important role in the regulation of inflammatory processes. They do not only secrete pro-inflammatory mediators (e.g., IL-6, TNF-α), thus triggering and maintaining the inflammatory response, but they also secrete anti-inflammatory mediators (e.g., IL-10), which terminate the inflammatory response [8,26]. In the present study, coixol inhibited the secretion of IL-1β, IL-6 and to a lesser degree that of TNF-α, but had no significant effect on the expression of IL-10, which suggests that pretreatment of coixol can inhibit the trigger and maintenance of inflammation. The activation of the ERK pathway is a common requirement of IL-10 expression in macrophages. However, the current study demonstrated that coixol inhibited the activation of the ERK pathway. Therefore, we speculated that post-transcriptional regulation may be the reason why IL-10 expression is not affected [27]. Unlike IL-1β and IL-6, the secretion of TNF-α requires the catalysis of TNF-α - converting enzyme (TACE) [28]. Only a high concentration of coixol can reduce the expression of TNF-α which may be related to a low concentration of coixol not being able to affect the activity of TACE. Similar results have been reported in a previous study, where it was shown that ethanol extracts of coix inhibited LPS-induced macrophages to secrete IL-6 and TNF-α, and, similar to the results from this study, the down-regulatory effect of the extracts on IL-6 was better than that on TNF-α [25]. We believe that these changes are the reason for the anti-inflammatory action of coixol. In addition, pretreatment of coixol inhibited the expression of NO and iNOS induced by LPS, which is another manifestation of its anti-inflammatory effect. It is known that pro-inflammatory mediators promote the synthesis of NO through inducible nitric oxide synthases (iNOS), and NO can directly activate the NF-κB pathway and induce the production of pro-inflammatory mediators, thus forming a positive feedback loop and maintaining inflammatory processes [29]. Coixol may also be able to alleviate the pain caused by inflammation because we have shown that pretreatment of coixol inhibited the expression of COX-2. COX-2 is a key regulatory enzyme for prostaglandin synthesis [30]. Prostaglandin E2 is associated with pain caused by inflammation [31]. Collectively, the coixol-induced regulation of inflammatory mediators including cytokines, NO, iNOS, and COX-2 suggests that the anti-inflammatory effect of coixol is achieved by down-regulating the expression of proinflammatory mediators.

In order to explore the possible mechanism of coixol-induced anti-inflammatory effects, the key kinases of NF-κB and MAPK pathways were examined. The phosphorylation of IκB and p65 signifies the degradation of IκB and the activation of NF-κB. The activated NF-κB translocates into the nucleus and upregulates the transcription of inflammatory genes, which is the classical model of NF-κB pathway activation [9]. Pretreatment of coixol inhibited the activation of the NF-κB pathway via the classical model. MAPK pathway regulates inflammatory genes via phosphorylation of ERK, JNK, and p38 [10]. In the current study, LPS-induced phosphorylation of ERK, JNK, and p38 was down-regulated by pretreatment of coixol. This result indicates that the activation of MAPK pathways was also inhibited. Previous evidence indicated the activated NLRP3 inflammasome not only directly promotes the maturation of pro-inflammatory factors (i.e., IL-1β and IL-18) but also regulates the production of pro-inflammatory factors by recruiting immune cells and mediating pyroptosis [11]. Upon activation, NLRP3 combine to ASC and subsequently induce the activation of procaspase-1. Procaspase-1 cleaves itself to caspase-1, which is required to convert pro-IL-1β to its mature active form. Coixol reduced the expression of three components of the NLRP3 inflammasome, NLPR3, caspase-1, and ASC. At the same time, IL-1β and IL-18, the products of the NLRP3 inflammasome activation, were also decreased. These results indicate that pretreatment of coixol inhibits the activation of the NLRP3 inflammasome. Activation of the NLRP3 inflammasome has been implicated in insulin resistance and associated diseases, such as Alzheimer’s disease, obesity, and cardiovascular disease [32,33,34]. Further in vivo studies are indicated to investigate if coixol would be useful as a therapeutic anti-inflammatory agent. So far, the highest dosage of coixol in reported in vivo studies was 50 mg/kg (about 300 μM/kg) [24]. However, whether this concentration shows certain toxicity was not mentioned. The concentration of coixol in the current study was higher than that in other in vitro studies [23,24]. The concentration difference may be related to the type of cells. Therefore, in consideration of the potential toxicity, the absorption, distribution, metabolism, and excretion of high dosage coixol in animals remains to be studied.

In conclusion, the present study confirmed that pretreatment of coixol can inhibit the secretion of pro-inflammatory mediators in LPS-induced macrophages. This effect of coixol is in part related to its inhibition of NF-κB, MAPK pathways, and NLRP3 inflammasome activation.

## 4. Materials and Methods 

### 4.1. Reagents

LPS was purchased from Sigma (Sigma-Aldrich, St. Louis, MO, USA). Coixol was purchased from Yuanye Bio-Technology (Yuanye Bio-Technology, Shanghai, China). NO assay kit (S0021), BCA protein assay kit (P0012), and bovine serum albumin (ST023) were obtained from Beyotime (Beyotime Biotechnology, Shanghai, China). ELISA kits for IL-1β (432604), IL-6 (431304), TNF-α (430904), and IL-10 (431414) were obtained from BioLegend (BioLegend, San Diego, CA, USA). Antibodies against iNOS and COX-2 were purchased from Affinity Biosciences (Affinity Biosciences, Zhenjiang, China). Antibodies against pIκB, pp65, GAPDH, and β-tubulin were purchased from Abcam (Abcam, Cambridge, UK). Antibodies against pERK, ERK, pJNK, JNK, pp38, and p38 were purchased from CST (Cell Signaling Technology, Bervely, MA, USA). Hypersensitive ECL chemiluminescence Kit (WLA006) and antibodies against NLRP3, ASC, Caspase-1, and IL-18 were purchased from Wanleibio (Wanleibio, Shenyang, China). Radio-Immunoprecipitation Assay Lysis Buffer was obtained from Solarbio (R0010, Solarbio, Beijing, China).

### 4.2. Cell Culture and Viability Assay

RAW264.7 cells were purchased from the Chinese Academy of Medical Sciences Peking Union Medical College (National Infrastructure of Cell Line Resource, Beijing, China). They were cultured in an incubator (MCO-5AC, Sanyo, Osaka, Japan) at 37 °C under a humidified atmosphere of 5% carbon dioxide. The maintenance medium was high glucose Dulbecco’s Modified Eagle’s Medium (DMEM, HyClone, Logan, UT, USA) containing 10% fetal bovine serum (FBS, Gibco, Grand Island, NY, USA) and no antibiotics.

Cell viability was measured using a Cell Counting Kit-8 (Dojindo, Shanghai, China). Briefly, cells were cultured in 96 well plates (5 × 10^4^ cells/well, 100 μL medium/well) for 12 h. After that, cells were incubated with various concentrations of coixol for 4 h. Thereafter, the supernatant was removed, CCK8 reagent was added and incubated in the incubator for 1 h. Finally, the absorbance of each well was measured at 450 nm using a microplate reader (Multiskan3, Thermo Fisher, San Diego, CA, USA).

### 4.3. Determination of NO Production

After overnight culture in 96 well plates (1 × 10^5^ cells/well, 100 μL medium/well), cells were pre-treated with various concentrations of coixol for 4 h, followed by a 4 h incubation with LPS. Then, the supernatant from each well was collected. The NO production was measured according to the manufacturer’s recommendations.

### 4.4. Determination of IL-1β, IL-6, TNF-α and IL-10 Production

After overnight culture in a 96 well plates (1 × 10^5^ cells/well, 100 μL medium/well), cells were pre-treated with various concentrations of coixol for 4 h, followed by a 4 h incubation with LPS. Then, the supernatant from each well was collected. ELISA kits were used to detect the concentrations of IL-1β, IL-6, TNF-α, and IL-10 according to the manufacturer’s recommendations.

### 4.5. Western Blotting

After overnight culture in 6 well plates (1 × 106 cells/well, 2000 μL medium/well), cells were pre-treated with various concentrations of coixol for 4 h and LPS for an additional 4 h. Then, the cells were harvested for western blotting. Radio-Immunoprecipitation Assay Lysis Buffer was used to lyse the cells on ice, and the lysate was centrifuged (14,000 rpm, 3 min), then each supernatant was collected. BCA protein assay kit was used for protein quantification. Protein samples were separated by SDS gel electrophoresis and electrotransferred onto polyvinylidene difluoride membrane. The membrane was sealed with 3% bovine serum albumin TBST (Tris-buffered saline with 0.1% Tween-20) solution, and then incubated with primary antibody overnight at 4 °C, washed with TBST 3 times and incubated for 1 h with peroxidase-conjugated secondary antibody at room temperature. After washing, a hypersensitive ECL chemiluminescence kit and chemiluminescence imaging system were used for detecting the band intensities.

### 4.6. Statistical Analysis

Statistical analysis was performed with one-way ANOVA followed by Bonferroni test in GraphPad Prism 5. The normal distribution of data was checked with Kolmogorov-Smirnov test in SPSS 20.0. The data were presented as mean ±standard deviation (SD). Differences with *p* < 0.05 were considered statistically significant. 

## 5. Conclusions

The present study revealed that coixol inhibits the activation of NF-κB, MAPK pathways, and NLRP3 inflammasome, down-regulates the expression of pro-inflammatory meditators IL-1β, IL-6, IL-18, TNF-α, NO, iNOS, and COX-2, and exerts anti-inflammatory effect. From these findings, we conclude that coixol has the potential to be a candidate for the development of therapeutic anti-inflammatory agents.
